# Genetic associations in community context: a mixed model approach identifies a functional variant in the *RBP4* gene associated with HDL-C dyslipidemia

**DOI:** 10.1186/s12881-018-0719-1

**Published:** 2018-11-29

**Authors:** Erfan Aref-Eshghi, Oliver Hurley, Guang Sun, Alvin Simms, Marshall Godwin, Pauline Duke, Mehdee Araee, Masoud Mahdavian, Shabnam Asghari

**Affiliations:** 1 Faculty of Medicine, Memorial University of Newfoundland, M5M107 Medical Education Building, 300 Prince Philip Drive, St. John’s, NL A1B 3V6 Canada; 2 Department of Geography, Memorial University of Newfoundland, St. John’s, NL Canada

**Keywords:** HDL, Dyslipidemia, RBP4, Mixed model, Genetics, Associations, SNP, Newfoundland

## Abstract

**Background:**

The objective of this study was to examine individual and community factors that influence high-density lipoprotein cholesterol (HDL-C) dyslipidemia in Newfoundland and Labrador (NL), a genetically isolated population in Canada with a high prevalence of HDL-C dyslipidemia.

**Methods:**

First, a group of single nucleotide polymorphisms from 10 metabolic trait candidate genes was tested using a multivariate logistic regression model. The significant SNPs were entered into the second phase, where a mixed logistic model incorporated the community disease risk factors together with the individual factors as the fixed part of the model and the geographic region as a random effect.

**Results:**

Analysis of 1489 subjects (26.9% HDL-C dyslipidemia) identified rs3758539, a non-coding variant in the 5’UTR of *RBP4*, to be associated with HDL-C dyslipidemia (odds ratio = 1.45, 95% confidence interval = 1.08–1.97, *p* = 0.01). The association remained significant, and the effect size did not change after the incorporation of individual and community risk factors from 17 geographic regions (odds ratio: 1.41, 95% confidence interval = 1.03–1.93, *p* = 0.03) in NL. Besides this variant, sex, BMI, and smoking also showed significant associations with HDL-C dyslipidemia, whereas no role was identified for the community factors.

**Conclusions:**

This study demonstrates the use of community-level data in a genetic association testing. It reports a functional variant in the promoter of *RBP4*, a gene directly involved in lipoprotein metabolism, to be associated with HDL-C dyslipidemia. These findings indicate that individual factors are the main reason for a higher prevalence of HDL-C dyslipidemia in the NL population.

## Background

Low serum levels of High-Density Lipoprotein Cholesterol (HDL-C) is the most common lipid abnormality found among patients with coronary artery disease [1, 2]. HDL-C dyslipidemia is a complex trait resulting from the cumulative and interactive effects of numerous genomic variants and environmental factors. It has a strong genetic component, such that through twin studies its heritability has been estimated to be around 40–60% [3]. Our previous study has shown that the prevalence of dyslipidemia of HDL-C is 11% higher in the Newfoundland and Labrador (NL) province of Canada compared to the rest of the country (38% vs. 27%) [4, 5]. It is not clear what causes an increased rate of HDL-C dyslipidemia in this population; however, the unique population history of NL can lead to clues as to what factors might be involved.

The majority of today’s NL population descend from 20,000 migrants who arrived in NL from England and Ireland in the mid-1700s [6]. This founding population experienced a low rate of in-migration over centuries, which resulted in NL to be one of the few remaining isolated founder Caucasian populations worldwide. As a result, the NL population has a unique cultural and genetic background, both of which can contribute to a higher rate of dyslipidemia. This makes NL a unique population for studying both the genetic and environmental factors that contribute to a human complex trait, e.g., dyslipidemia. In population genetics terms, NL has been shown to encompass a reduced rate of heterozygosity, increased runs of homozygosity, and extended genomic linkage disequilibrium [6, 7]. This founder population, however, does not have an entirely homogenous genetic structure, and according to the religion (i.e., Catholic vs. Protestant) and the ethnicity of the founding groups (i.e., British vs. Irish) which tended to reside and remain in different geographical areas, the current NL population can be grouped into multiple clusters [7]. This geographic variability in the NL’s population structure enables studying, simultaneously, both individual- and community-based factors that contribute to a complex human trait.

Traditional genetic studies of complex traits use epidemiological approaches to search for the association of genetic variants with the trait of interest. Such studies are performed on an individual level basis, and often the environment in which the individual resides is not considered [8, 9]. Mounting evidence suggests that an individual’s health is directly influenced by the community health indices of the society they live in [10, 11], including the community’s disease status, availability of health care and treatment, and the level of exposure to hazards. Therefore, a study that takes into account both individual- and geographical/community-level data may add more to the mechanism of involvement of a candidate genetic variant or other risk factors in a complex trait, e.g., HDL-C dyslipidemia.

Mixed models have been introduced as an advanced analytical tool to model the population stratification and unequal relatedness among individuals in genetic association studies [12]. The present study utilized a mixed model design to examine the individual (genetic and non-genetic) and community factors that contribute to a higher rate of HDL-C dyslipidemia in the NL population.

## Material and methods

### Source of data

We conducted a secondary analysis of individual and community level data from three existing databases. The study only included individuals over 20 years of age living in NL at the time of blood test, excluding pregnant women. The first dataset was only used to obtain individual-level data, whereas the other two datasets provided the community-level data. These data sources include:

1. COmplex DIseases in the Newfoundland population, environment, and Genetics (CODING) study: this ongoing study explores genetic, endocrine and nutritional links to metabolic traits in NL [13]. At the time of the study, over 3200 third generation Newfoundlanders participated in the program. Recruitment criteria were as follows: (i) at least third generation Newfoundlander; (ii) healthy, without any serious metabolic, cardiovascular or endocrine disease; and (iii) not pregnant at the time of participation. Extensive measurements including a complete lipid profile, history of diabetes, smoking, blood pressure and body mass index (BMI) were completed for these participants. All participants provided blood samples following a 12-h fasting period. Serum was isolated for measuring the concentrations of lipoproteins using Synchron reagents, which was performed on an Lx20 analyzer (Beckman Coulter, Fullerton, CA). Additionally, genotyping of 40 single nucleotide polymorphisms (SNPs) from 10 genes associated with metabolic traits was performed for a subset of the cohort. Tagging SNP selection was done according to the linkage disequilibrium (LD) block patterns of every gene to maximize the gene coverage using a pairwise R-squared approach (SNPbrowser Version 3.5; Applied Biosystems; based on HapMap data for CEU population). Genomic DNA was isolated from ∼5 ml of whole blood using the Wizard Genomic DNA Purification kit (Promega, Madison, WI). Genotyping was performed using Taqman validated or functionally tested SNP Genotyping Assays (Applied Biosystems, Foster City, CA) on an ABI Prism 7000 Sequence Detection System (Applied Biosystems). All experiments were conducted according to the manufacturers’ protocols. To assess the reproducibility of genotyping, 5% of the samples were randomly selected and re-genotyped, all of which were confirmed to match their original called genotypes. Only subjects for whom complete genotyping was performed were included in the analysis.
2. Newfoundland and Labrador Laboratory Information System (LIS) Data: for each laboratory service provided in NL, the patient’s identification, date of service, and laboratory test results are entered into this database. Therefore, this dataset represents all of the HDL-C measures that were reported by the clinical laboratories across the province. From this dataset, we obtained all of the HDL-C test results performed in NL during January 2011 and December 2011, along with the sex of the patients. The year 2011 was chosen because it encompassed the most comprehensive data across all of the three databases in this study. It was also the year in which the majority of the CODING study participants were recruited.
3. Digital Epidemiology Chronic Disease Tool (DEPICT) Data: DEPICT is a spatiotemporal information system in NL which collects disease and demographic information on adults with chronic diseases from provincial medico-administrative data. For this study, we obtained population age and prevalence of diabetes and hypertension in the year 2011.

HDL-C dyslipidemia in both the CODING and LIS datasets was defined as HDL-C levels ≤1.0 mmol/dl for males and ≤ 1.3 mmol/dl for females according to the Canadian guidelines for the diagnosis and treatment of dyslipidemia [14].

### Determination of geographic/community level

For community-level analysis, the collected data was aggregated by a geographic stratifier. The level of geography to be used for this purpose was determined by examining the ratio of individuals included in the above datasets in each existing geographic unit in NL to the total population count living in that region. Canadian postal codes are composed of six alphanumeric characters, the first three of which are called the forward sortation area (FSA). Using sensitivity analysis we found that FSA has the best distribution of the records across the three data sources, providing the most optimal representation of NL’s population distributions. Therefore, the variables from the community-level data (DEPICT and LIS) were aggregated by FSA.

### Statistical analysis

Individuals with HDL-C dyslipidemia were compared with those with normal levels of HDL-C. The analysis was performed in two steps:

1. The first step was conducted on the CODING study data only at the individual level. All of the SNPs were tested for the Hardy-Weinberg Equilibrium among the control group using a Pearson chi-squared test, and those with a *P*-value< 0.01 were determined to be deviating from the equilibrium and were excluded. With the assumption of an additive genetic effect, a multivariable logistics regression model was fit between HDL-C dyslipidemia and every SNP. The genotypes were coded as 0, 1 and 2, representing individuals with zero, one, and two copies of the risk allele for every SNP to allow for testing the association between the SNPs and HDL-C dyslipidemia. Individual factors related to dyslipidemia including sex, age, smoking, BMI, hypertension, and diabetes were treated as covariables in the model. Using the median values as cut-offs, these continuous variables were transformed to binary categorical variables to improve the model’s goodness of fit. Male was considered as the base for the sex variable.
2. The SNPs that resulted in a *p*-value of less than 0.05 were used in the second step of the analysis. In this step, a multilevel logistic regression modeling was performed. The individual-level variables from the CODING study along with the community-based factors from the LIS and DEPICT were fit as the fixed effect, while the community classification code (FSA) together with the same community factors from LIS and DEPICT were incorporated as the random effects. The community-based data from these two datasets were obtained by aggregating the variables on the FSA level. These include mean population age and sex compositions, as well as the rate of HDL-C dyslipidemia, hypertension, and diabetes in every FSA unit. The individual level HDL-C dyslipidemia from CODING was treated as the binary outcome. Residual plots of the fitted models were inspected to ensure no deviation from normality or homoscedasticity was present. The analysis was performed using the *lme4* package in R 3.3.2 [15].

## Results

### Data descriptions

The CODING dataset was composed of 962 males and 2358 females aged 42.6 ± 13.2 with a mean BMI of 26.7 ± 5.2. Among this population, 4.0% were diabetic, 32.6% had hypertension, and 15.3% were smokers. The prevalence of HDL-C dyslipidemia in this cohort was 26.9%. The SNP genotyping was performed for 1489 subjects, who were included in the analysis. Characteristics of the healthy and dyslipidemic groups are presented in Table 1. The risk allele, its frequencies among those with and without HDL-C dyslipidemia and the overlapping genes are shown in Table 2 for every SNP.

**Table 1:** Individual characteristics of CODING study with/without HDL-C dyslipidemia

Individual characteristics	Normal HDL-C (*n* = 1191)	HDL-C Dyslipidemia (*n* = 298)
Age^a^	42.3 ± 11.3	43.3 ± 10.9
Sex (F)	75.7%	85.9%
BMI^a^	26.1 ± 4.6	29.2 ± 5.8
Smoking	11.7%	16.6%
Diabetes	2.9%	7.1%
Hypertension	26.3%	31.1%

^a^Mean ± standard deviation

**Table 2:** SNP characteristics and multivariate logistics regression analysis

Gene Symbol	SNP	Alleles	Risk Allele	Risk Allele Frequency		Odds Ratio (95% Confidence Interval)	*P*-value
				Normal HDL-C	Abnormal HDL-C		
*ADFP*	rs35629534	A/G	A	0.95	0.96	1.22 (0.74–2.13)	0.464
*ADFP*	rs3824369	C/T	C	0.49	0.51	1.10 (0.89–1.37)	0.369
*ADIPOQ*	rs1063537	C/T	C	0.90	0.92	1.23 (0.88–1.76)	0.245
*ADIPOQ*	rs182052	A/G	A	0.33	0.36	1.11 (0.90–1.37)	0.325
*ADIPOQ*	rs6773957	A/G	A	0.38	0.37	0.99 (0.81–1.22)	0.941
*ATGL*	rs1138714	A/G	A	0.45	0.47	1.14 (0.92–1.40)	0.223
*CART*	rs3763154	T/C	T	0.57	0.58	1.06 (0.87–1.30)	0.555
*CART*	rs2320167	A/G	A	0.10	0.11	1.17 (0.84–1.60)	0.344
*GHRL*	rs35684	A/G	A	0.73	0.76	1.22 (0.97–1.55)	0.089
*GHRL*	rs2075356	C/T	C	0.11	0.11	1.02 (0.74–1.39)	0.910
*GHRL*	rs26311	C/G	C	0.88	0.86	0.87 (0.65–1.17)	0.344
*GHRL*	rs4684677	A/T	A	0.06	0.07	1.01 (0.66–1.51)	0.971
*GHRL*	rs26802	G/T	G	0.30	0.28	0.91 (0.73–1.14)	0.409
*MCHR1*	rs882111	G/T	G	0.93	0.92	0.93 (0.64–1.37)	0.698
*MCHR1*	rs9611386	A/G	A	0.95	0.95	0.95 (0.62–1.52)	0.837
*MCHR*	rs133074	C/T	C	0.50	0.51	1.07 (0.88–1.31)	0.488
*MCHR*	rs133073	C/T	C	0.42	0.41	0.91 (0.74–1.11)	0.354
*PLIN*	rs4932241	A/C	A	0.31	0.30	0.91 (0.72–1.16)	0.459
*PLIN*	rs894160	C/T	C	0.74	0.72	0.85 (0.67–1.09)	0.190
*PLIN*	rs2289487	C/T	C	0.31	0.33	1.15 (0.91–1.45)	0.253
*RBP4*	rs3758539	C/T	C	0.83	0.87	1.45 (1.08–1.96)	0.015*
*RBP4*	rs779604024	A/G	A	0.91	0.91	1.12 (0.79–1.62)	0.544
*RBP4*	rs10882280	A/G	A	0.09	0.09	0.94 (0.65–1.33)	0.720
*TTS2.2*	rs4131364	A/G	A	0.50	0.48	0.90 (0.73–1.11)	0.308
*NAMPT*	rs7789066	A/G	A	0.94	0.93	0.92 (0.61–1.41)	0.689
*NAMPT*	rs6947766	C/T	C	0.74	0.76	1.12 (0.89–1.42)	0.326
*NAMPT*	rs6963243	C/G	C	0.24	0.27	1.22 (0.97–1.54)	0.093
*NAMPT*	rs3801266	C/T	C	0.19	0.17	0.87 (0.66–1.14)	0.332
*NAMPT*	rs10808150	A/G	A	0.43	0.43	1.05 (0.85–1.29)	0.648
*NAMPT*	rs2098291	C/T	C	0.69	0.68	0.96 (0.77–1.19)	0.684
*PLA2G16*	rs7101608	G/C	G	0.45	0.49	1.14 (0.94–1.39)	0.194

*P-value< 0.05; All analyses were adjusted for age, sex, BMI, smoking, and comorbidities (diabetes and hypertension)

The LIS dataset contained 121,090 records. The mean age was 56.0 ± 14.1 years, and 23.1% were classified as having HDL-C dyslipidemia. DEPICT data was composed of 226,530 electronic records (52.0% females). Table 3 shows this information for every FSA region.

**Table 3:** Population variations in community risk factors, obtained from LIS and DEPICT

FSA	Count (Total = 1230)^ab^	Total Population^d^	HDL-C Dyslipidemia Prevalence^c^	Population Proportion of Females^d^	Average Population Age^b^	Hypertension Prevalence^d^	Diabetes prevalence^d^
A0A	74	36,885	0.22	0.50	46.2	0.11	0.29
A0B	16	17,315	0.22	0.50	50.8	0.10	0.14
A0H	7	14,890	0.48	0.54	48.3	0.10	0.00
A1A	202	23,270	0.19	0.52	40.9	0.10	0.17
A1B	123	14,490	0.20	0.53	32.9	0.07	0.16
A1C	63	11,380	0.20	0.52	39.2	0.09	0.18
A1E	202	23,215	0.21	0.55	43.5	0.09	0.20
A1G	20	4295	0.24	0.53	40.8	0.09	0.11
A1H	5	2415	0.19	0.51	34.5	0.07	0.12
A1K	73	8335	0.20	0.50	38.4	0.10	0.15
A1L	74	13,110	0.20	0.52	35.2	0.07	0.11
A1M	44	5520	0.21	0.49	39.5	0.11	0.19
A1N	217	19,215	0.22	0.52	41.6	0.09	0.19
A1S	26	4955	0.23	0.49	40.3	0.09	0.13
A1V	5	8475	0.27	0.55	41.1	0.12	0.25
A1W	43	8380	0.21	0.49	40.3	0.09	0.15
A1X	36	10,385	0.22	0.50	39.5	0.11	0.27

^a^259 individuals had no postal codes or resided in areas with less than a total sample of 5 individuals and therefore were excluded from the initial 1489 cohort. ^b^CODING data; ^c^LIS Data; ^d^DEPICT data

### Identification of the genetic variants associated with HDL-C dyslipidemia

This phase was performed on individual-level data from the CODING cohort. All of the available SNPs (*n* = 40) were queried using the SNP Annotation and Proxy search tool (SNAP Version 2.2; BROAD Institute, Cambridge, Massachusetts, US) for pair-wise LD measurements, eight of which were found to be in high LD (r^2^ > 0.8) with the others. Following the removal of these 8 SNPs, 32 SNPs were included in the analysis. All SNPs had a minor allele frequency above 5%. One of the SNPs deviated from Hardy-Weinberg Equilibrium and thus was excluded from the analysis. Among the remaining 31 SNPs, only one, rs3758539, located in *RBP4*, obtained a *p*-value < 0.05 (odds ratio: 1.45, 95% confidence interval: 1.08–1.97, p-value: 0.01). The risk allele frequency of this SNP was 4% higher in the dyslipidemia group compared with the healthy group (87% vs. 83%). Other co-variables which showed a significant association with HDL-C dyslipidemia were female sex (odds ratio: 2.25, 95% confidence interval: 1.52–3.41, p-value: 7.86e-05), BMI (odds ratio: 1.12, 95% confidence interval: 1.09–1.15, p-value: 3.30e-16), and smoking (odds ratio: 1.71, 95% confidence interval: 1.15–2.50, *p*-value: 0.006). The complete results of the analyses for these 31 SNPs are shown in Table 2.

### Identification of individual and community factors influencing HDL-C dyslipidemia

**Fig. 1:**
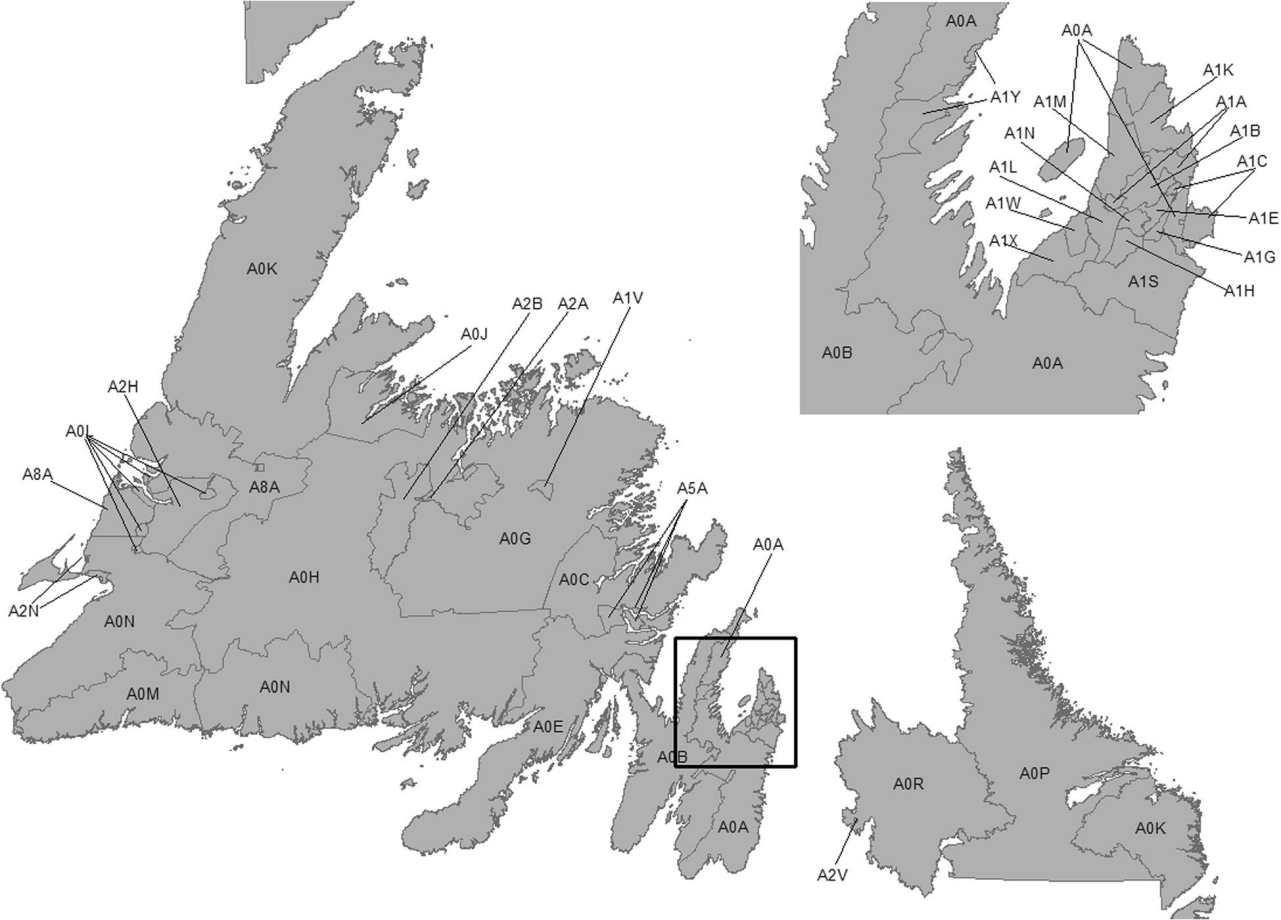
FSA codes in Newfoundland and Labrador. The square represents the region included in the study, where the majority of NL’s population resides

The second phase of the study linked the LIS and DEPICT datasets to the CODING data using the geographic identifier (FSA). The postal code information was available for about 80–85% of the subjects in the CODING dataset. NL is composed of 35 FSA regions (Fig. 1). The total number of FSA regions with at least five records in the CODING data for every variable was 17. Table 3 shows the level of variation in the community-level data as well as the risk allele frequency of rs3758539 across these 17 FSA regions. These results indicate a degree of variability for the calculated indices across these FSAs.

The inclusion of community-level factors was performed in two steps. First, only the geographic identifier was included as a random effect into the original model fitted earlier, which did not significantly change the parameters of the observed associations (Table 4), indicating that the variability between different communities for these factors is minimal. Next, the community-level factors were added as both the fixed effect and the random effect to the model. It was observed that the effect of these community factors on the individual factor associations is also negligible (Table 4). None of the community-level factors showed any association with HDL-C dyslipidemia or affected its association with individual-level factors. The analysis was also repeated after removal of the community-level factors from the random effect, which produced a similar result (data not shown). This final model indicated that only individual factors of sex (Odds Ratio: 2.10; 95% confidence interval: 1.36–3.23), BMI (Odds Ratio: 1.12; 95% confidence interval: 1.08–1.15), and smoking (Odds Ratio: 1.69; 95% confidence interval: 1.12–2.55) besides rs3758539 influence HDL-C dyslipidemia.

**Table 4:** Multilevel modeling of individual and community risk factor associations with HDL-C dyslipidemia

	Model 1: Intercept only		Model 2: + Individual variables		Model 3: + Community variables	
	Odds Ratio (95% Confidence Interval)	*p*-value	Odds Ratio (95% Confidence Interval)	*p*-value	Odds Ratio (95% Confidence Interval)	*p*-value
Fixed Effects						
Intercept	0.39 (0.34–0.44)	< 0.0001	0.003 (0.001–0.009)	< 0.0001	0.01 (0.01–0.13)	< 0.0001
Individual-level variables						
Sex (M = 0, F = 1)			2.12 (1.38–3.26)	0.0006	2.10 (1.36–3.23)	0.0007
Age (years)			1.00 (0.99–1.02)	0.5	1.00 (0.99–1.02)	0.4
BMI			1.12 (1.08–1.15)	< 0.0001	1.12 (1.08–1.15)	< 0.0001
Diabetes			1.28 (0.63–2.61)	0.5	1.29 (0.63–2.64)	0.5
Hypertension			0.99 (0.70–1.41)	0.9	0.97 (0.68–1.38)	0.8
Smoking			1.66 (1.10–2.51)	0.01	1.69 (1.12–2.55)	0.01
rs3758539			1.40 (1.03–1.92)	0.03	1.41 (1.03–1.93)	0.03
Community-level variables						
Prevalence of HDL dyslipidemia					0.52 (0.03–8.63)	0.6
Population Proportion of Females					0.84 (0.54–1.31)	0.4
Population Mean Age					0.73 (0.47–1.12)	0.1
Prevalence of Hypertension					0.96 (0.54–1.69)	0.9
Prevalence of Diabetes					0.83 (0.52–1.31)	0.4
Random Effects^a^						
Community classification code (FSA)		0.02	< 0.0001		< 0.0001	
Prevalence of HDL dyslipidemia					< 0.0001	
Population Proportion of Females					< 0.0001	
Population Mean Age					< 0.0001	
Prevalence of Hypertension					< 0.0001	
Prevalence of Diabetes					< 0.0001	

^a^Values represent variance

## Discussion

This study attempted to use a multilevel modeling approach to identify individual and community level factors that influence HDL-C dyslipidemia in NL, a genetically isolated population. Mainstream studies of individual, genetic or community factors in complex traits do not combine these lines of investigations despite their interactive role in the development of complex conditions. This is particularly important in genetic association testing where the majority of the reported genetic associations are known to be population specific, and replication rates are as low as 5% [16, 17], despite controlling for population structures. One reason for this, which has not been investigated enough, might be the variations in the community disease risks across different populations and/or subpopulations. We used a mixed model approach to model the community factors into the association between genetic variants and HDL-C dyslipidemia. Mixed models in genetic association studies have the advantage of preventing false-positive findings due to population or family-relatedness structures and increasing the power by applying a correction to them [18]. As such, this approach would have increased the power in our study by correcting for sub-population variations in the community risk factors of HDL-C dyslipidemia. Our study, however, did not find that community factors can significantly influence the association between a SNP and HDL-C dyslipidemia. Yet, this should not lead to the conclusion that community-level factors do not alter the associations of genetic or non-genetic factors in complex conditions, since we did not test the multitude of other genomic loci associated with dyslipidemia or did not extend the study to other complex traits.

Our study found that the risk allele (C) of SNP rs3758539 (C > T), adjusted for other individual risk factors, is associated with ~ 1.40 increased odds of having HDL-C dyslipidemia, and this association remains significant when other individual- or community-based factors are taken into account. This SNP has a potential to be a causal variant in HDL-C dyslipidemia. rs3758539 is located in the 5’UTR of the Retinol Binding Protein 4 (*RBP4*) gene. This region is an active promoter and harbors an enhancer element. A query in HaploReg v4.1 database (BROAD Institute, Cambridge, Massachusetts, US) reveals that it encompasses regulatory chromatin features and is in high LD (r^2^ > 0.8) with multiple other functional SNPs. In vitro studies show that alternative alleles of this variant induce a differential transcriptional activity and variable binding affinities to the transcription factor hepatocyte nuclear factor 1 alpha (HNF1α) [19]. The minor allele (T) is specifically shown to be associated with a higher expression of *RBP4* in adipocytes [20]. Multiple sequence alignments of human, mouse, rat, and cattle *RBP4* promoter also indicates that the SNP is within a conserved element harboring seven transcription factor binding motifs [20]. Human genetic studies suggest that rs3758539 is associated with an increased risk of type 2 diabetes [19] and obesity [21]. Another SNP (rs12265684), which is in a tight LD with rs3758539, is associated with serum levels of triglycerides, and insulin resistance [22]. The present study will be the first report to directly associate this variant with HDL-C dyslipidemia. A previous study comparing 304 lean and 283 obese Caucasian children found a slight increase in the risk allele of this SNP in the obese group and those having more undesirable cardiometabolic parameters including dyslipidemia, being consistent with the findings of our study; however, their analysis failed to produce a significant *p*-value (*p* = 0.3) likely due to insufficient sample size [23]. Another study in a Chinese cohort did not identify any association between HDL-C levels and rs3758539 [24]. The same observation has been found in a second Chinese cohort [25]. These studies indicate that the observed association is most likely specific to the Caucasian population, yet not all ethnic groups have been explored. A previous study in NL population by Shea et al. [26] did not find an association between this variant and HDL-C serum levels. Instead, they found two other *RBP4* variants (rs10882280 and rs11187545) associated with the serum levels of HDL-C. These two SNPs are not in LD with our SNP (r^2^ = 0.01), but have a strong LD with each other (r^2^ = 1.0). We tested one of them in our database and did not observe an association with HDL-C dyslipidemia (Table 2). The putative reason for the discrepancy between these two studies might be related with designing of the outcome variables in the two studies (HDL-C serum levels vs. HDL-C dyslipidemia). HDL-C dyslipidemia is defined using different serum HDL-C cut-offs for males and females, and this gives a differential weight to males and females in statistical modeling, which cannot be resolved simply by adjusting for the sex co-variable. Regardless of which approach is the most appropriate, both studies identify *RBP4* as a potential candidate for HDL-C dyslipidemia.

The *RBP4* gene encodes a binding protein to retinol (vitamin-A alcohol) which is involved in preventing vitamin-A loss during glomerular filtration and delivering it from the liver stores to the peripheral tissues. It is assumed that RBP4 regulates lipid homeostasis by the activation of nuclear receptors through retinol metabolism [27]. This regulation has been under such scrutiny that recent studies have attempted to use RBP4 as an early marker of metabolic abnormalities including dyslipidemia [28, 29]. Serum levels of RBP4 have also been reported to be positively correlated with HDL-C serum levels [30]. This is consistent with our finding that the risk allele (C) of rs3758539, being associated with a lower expression of *RBP4* [20], confers a risk towards having HDL-C dyslipidemia. However, several studies have reported negative correlations between the serum levels of HDL-C and RBP4 [31, 32], and thus further research is warranted to assign a causal relationship between rs3758539 and HDL-C dyslipidemia.

Our data also identified female sex (odds ratio: 2.25) as the strongest associated factor with HDL-C dyslipidemia followed by smoking (odds ratio: 1.71) and BMI (odds ratio: 1.12). We also tested age and comorbidities, but no association was observed. Smoking and obesity are amongst the most well-known risk factors for HDL-C dyslipidemia. A higher rate of HDL-C dyslipidemia in females and smokers in this study is also consistent with our previous studies of the primary-care population in NL [33–36].

Low HDL-C level is a major risk factor for cardiovascular diseases, the third cause of death worldwide. Knowing the genes involved in its pathogenesis is the frontline of further research for designing therapeutic and diagnostic interventions. Our study, for the first time, reports the association of a functional SNP with HDL-C dyslipidemia using a mixed model analysis, which is not frequently conducted in genetic association studies. Our study can be regarded as a proof of principle for incorporation of a multi-level approach in genetic association testings.

This study is limited by a few factors. Conducting a community-based study requires a large sample size from every geographic location. This is particularly important in genetic studies of complex traits where the effect sizes are expected to be small. In several of our geographic FSA regions, the sample sizes were not significantly large, and it is possible that this obscured the community effect that we expected to observe. It should also be noted that genetic associations are not valid unless replicated. This should be taken into account particularly in the presence of moderate effect sizes.

## Conclusion

Using a multilevel modeling approach, this study identified a genetic variant with potentially functional attributes in the promoter of the *RBP4* gene to be associated with HDL-C dyslipidemia. We found that this association is robust and is not altered if the community factors are taken into account. Also, our analyses did not establish any associations between the community level factors and HDL-C dyslipidemia in the NL population. However, we observed a significant role for individual obesity and smoking in suboptimal HDL-C levels. These findings suggest that the individual genetic and lifestyle factors are the main reason for a higher prevalence of HDL-C dyslipidemia in the NL province of Canada.

## Abbreviations

CVD: Cardiovascular disease
HDL-C: High-density lipoprotein cholesterol
LDL-C: Low-density lipoprotein cholesterol
LIS: Laboratory information system
NL: Newfoundland and Labrador
OR: Odds ratio
SNP: Single nucleotide polymorphism
TC: Total cholesterol
TG: Triglyceride
